# Audiovisual emotional processing and neurocognitive functioning in patients with depression

**DOI:** 10.3389/fnint.2015.00003

**Published:** 2015-01-30

**Authors:** Sophie Doose-Grünefeld, Simon B. Eickhoff, Veronika I. Müller

**Affiliations:** ^1^Department of Clinical Neuroscience and Medical Psychology, Medical Faculty, Heinrich Heine University DüsseldorfDüsseldorf, Germany; ^2^Institute of Neuroscience and Medicine, Research Centre JülichJülich, Germany

**Keywords:** depression, emotional processing, neurocognitive functioning, audiovisual, executive deficits, congruent, symptom severity

## Abstract

Alterations in the processing of emotional stimuli (e.g., facial expressions, prosody, music) have repeatedly been reported in patients with major depression. Such impairments may result from the likewise prevalent executive deficits in these patients. However, studies investigating this relationship are rare. Moreover, most studies to date have only assessed impairments in unimodal emotional processing, whereas in real life, emotions are primarily conveyed through more than just one sensory channel. The current study therefore aimed at investigating multi-modal emotional processing in patients with depression and to assess the relationship between emotional and neurocognitive impairments. Fourty one patients suffering from major depression and 41 never-depressed healthy controls participated in an audiovisual (faces-sounds) emotional integration paradigm as well as a neurocognitive test battery. Our results showed that depressed patients were specifically impaired in the processing of positive auditory stimuli as they rated faces significantly more fearful when presented with happy than with neutral sounds. Such an effect was absent in controls. Findings in emotional processing in patients did not correlate with Beck’s depression inventory score. Furthermore, neurocognitive findings revealed significant group differences for two of the tests. The effects found in audiovisual emotional processing, however, did not correlate with performance in the neurocognitive tests. In summary, our results underline the diversity of impairments going along with depression and indicate that deficits found for unimodal emotional processing cannot trivially be generalized to deficits in a multi-modal setting. The mechanisms of impairments therefore might be far more complex than previously thought. Our findings furthermore contradict the assumption that emotional processing deficits in major depression are associated with impaired attention or inhibitory functioning.

## INTRODUCTION

Major depression is a psychiatric disorder that is thought to represent one of the leading causes of disability worldwide (Ferrari et al., 2013). The disorder goes along with a range of symptoms including on the one hand emotional and social problems like low mood and loss of self-esteem as well as on the other hand cognitive impairments like poor concentration and indecisiveness (WHO, 2010). With regard to the former symptoms several theories have been postulated in order to gain a better understanding of the origins of these social and emotional problems in depression. The most influential theory suggests a negative bias for emotional but also neutral material, manifesting, for example, as more negative ratings of facial expressions or selective attention on negative stimuli. This has been supported by numerous studies (Gur et al., 1992; Bouhuys et al., 1999; Leppänen et al., 2004) reporting either a general negative bias or a bias specifically for neutral or ambiguous stimuli. However, most of these studies primarily investigated photographs or even schematic paintings of faces depicting emotions or neutrality (for a review, see Bourke et al., 2010). More recent studies now included on the one hand facial stimuli with varying intensity levels (Schaefer et al., 2010), and on the other hand other kinds of stimuli addressing different emotional channels such as voices (Schlipf et al., 2013) or music (Naranjo et al., 2011). Among these, fewer study outcomes indicated a clear negative bias in depression, but also an absence of a “healthy” positive bias. That is, while healthy non-depressed controls tend to interpret stimuli as positive, patients with depression do not. Joormann and Gotlib (2006) for example showed that when identifying emotions from faces, depressed individuals compared to controls needed significantly higher emotional intensity in order to correctly identify happy but not sad facial expressions. In addition, Loi et al. (2013) also only found labeling problems for happy body language depicted by photographs of body postures as well as frozen movie scenes and short clips of “Point-Light Walkers.” Although Schlipf et al. (2013) report a negative bias in reference to judgments of neutral semantics, patients also rated positive semantics and positive prosody as less positive than healthy controls, thus also indicating an absence of a positive bias in the patient group.

To date, most previous studies have investigated emotional processing using only unimodal stimuli. In daily life, though, emotions are hardly ever conveyed through just one sensory modality but rather in a multimodal fashion, i.e., seeing a happy facial expression and concurrently hearing the sound of laughter. Thus, it is in question if findings from unimodal emotional processing reflect deficits in real life. However, there are very few studies, which used stimuli from more than just one modality. Schneider et al. (2012) for example presented short video clips of actors conveying emotions via facial expressions, semantics and prosody and found patients to be impaired in recognizing emotions, but did not find an overall negative bias. In addition, in an earlier study (Müller et al., 2014), we investigated audiovisual emotional integration in major depression using functional magnetic resonance imaging. There we demonstrated that impairments in emotional processing in patients with depression seem to be far more complex than a simple bias as we found patients to be impaired in the inhibition of auditory stimuli presented with emotionally congruent facial expressions. However, owing to the relatively low sample size in that imaging study, there were only tendencies toward a behavioral effect. Therefore the current study aims at complementing the previous study by investigating multi-modal emotional processing (on the behavioral level) in an extended sample of patients with depression.

Furthermore, besides the postulation of emotional biases, there is also an ongoing discussion if alterations in emotional processing result from the likewise prevalent executive deficits in these patients. Executive functions, however, rather ill defined, include amongst others inhibition, working memory as well as cognitive flexibility (Diamond, 2013). Furthermore executive skills as the basis for everyday life functioning also include cognitive domains like attention (as a precondition for inhibitory functions; DeBattista, 2005). Impairments in the mentioned cognitive functions have been shown to be present in depression. Airaksinen et al. (2004), for example, reported deficits in cognitive flexibility in individuals with depression, and Rose and Ebmeier (2006) described impairments in working memory. Furthermore, attentional deficits have been found to be present even in remitted states of depression (Paelecke-Habermann et al., 2005). Interestingly, Hoffstaedter et al. (2012) reported significantly worse performance on verbal vocabulary testing in patients with depression compared to controls, and they related these impairments to memory deficits. In addition, they found group differences in attention, cognitive flexibility, (visuo-) motor coordination, short-term and working memory, but not for basic motor speed. Overall, psychomotor retardation, however, has been described as a core feature of depression (Sobin and Sackeim, 1997) and Hoffstaedter et al. (2012) were able to show that patients were impaired in specific cognitive aspects of psychomotor functioning.

Regarding the treatment of cognitive aspects of depression, Owens et al. (2013) were able to show that working memory training in dysphoric individuals can improve inhibition of irrelevant information and thus lead to increased working memory capacity. Since poor inhibitory control has been shown to be related to problems in the interpretation of emotional information in depression (Joormann, 2004; Goeleven et al., 2006), cognitive functioning seems to be a valuable starting point in the therapy of depressive symptoms. In line with this view, Marazziti et al. (2010) hypothesized that decreased cognitive flexibility in patients with depression possibly prevents those individuals from being able to cope with life events which then leads to constant low mood due to increased stress exposition. All in all, there is much evidence that depression goes along with impaired cognitive performance, and symptom severity seems to be related especially to decreased episodic memory, executive functions, and processing speed (McDermott and Ebmeier, 2009). Thus, it seems reasonable that deficits in emotional and cognitive processing might be closely interrelated. However, studies in depression investigating the relationship between deficits in emotional perception of faces and sounds and impairments in cognitive functions, measured independently from emotional processing, are rather rare. Taken together, even though findings clearly indicate an impairment in emotional processing in major depression, authors do not agree on whether depression is associated with a general bias (present negative or absent positive bias), if it is possibly a result of cognitive deficits, or both.

The aims of the current study were therefore to first assess uni- and multi-modal emotional processing in patients with depression. Second, we explored executive functioning and related cognitive domains to be able to investigate the potential relationship of emotional and cognitive deficits. We chose a selection of different neurocognitive tests where patients with depression have been reported to be impaired (Hoffstaedter et al., 2012), i.e., that challenged the participants’ attention, cognitive flexibility, (visuo-) motor speed and coordination, short-term as well as working memory, and verbal vocabulary.

Based on the previous literature regarding emotional processing in patients with depression, we hypothesized that for unimodal conditions, we would find a mood-congruent emotional bias in patients with depression (negative or absent positive), whereas in the multi-modal setting, impairments would probably appear in a manner different from a generalized bias as already described by Müller et al. (2014). Additionally we expected patients to perform worse than healthy controls on neurocognitive tests, especially those regarding cognitive flexibility and attention, and that these deficits would be associated with impairments in emotional processing, thus pointing in the direction that emotional problems in depression are related to likewise prevalent cognitive deficits.

## MATERIALS AND METHODS

### SUBJECTS

The current study is based on a previous study, which tested audiovisual emotional processing in depression by using fMRI (Müller et al., 2014). We now focus on the behavioral effects in an expanded sample of patients and healthy controls.

In total, 41 patients diagnosed with major depression (19 females, 22 males) and 41 healthy controls (19 females, 22 males) were now included. Data from 44 of these 82 subjects originated from the previous fMRI study, while the remaining 38 subjects conducted the paradigm outside the scanner. Both patients and controls gave informed consent into the study, which was approved by the ethics committee of the School of Medicine of the RWTH Aachen University. In addition to gender matching, the two groups did not differ in their age or years of education (age: *T*_80_ = -0.47, *p* = 0.64; EDU: *T*_80_ = 0.08, *p* = 0.80; see **Table 1** for means). All subjects were right-handed according to the Edinburgh Handedness Questionnaire (Oldfield, 1971) and had normal or corrected-to-normal vision. Patients were recruited from the inpatient and outpatient units of the Department of Psychiatry, Psychotherapy and Psychosomatics, RWTH University Hospital. They were diagnosed by their treating psychiatrist with a depressive episode or a recurrent depressive disorder according to the criteria of the ICD-10 (WHO, 2010; see **Table 2** for the patients’ clinical profiles). To confirm their diagnosis and to screen for possible psychiatric co-morbidities, the structured clinical interview for DSM-IV (SCID; Wittchen et al., 1997) was conducted. Furthermore, the Beck Depression Inventory (BDI-II; Hautzinger et al., 2006) as well as the Hamilton depression scale (Hamilton, 1960) were used to quantify depression-related symptoms and thus the illness severity. We only included patients without co-morbidities, i.e., without an indication of any psychiatric or neurological disease other than major depression, and without any kind of addiction or substance abuse in at least 6 months. Control subjects did not report any history of psychiatric or neurological disorders as well as any addiction in their past. Sub-clinical depressive symptoms in the control group were also assessed with the BDI-II (Hautzinger et al., 2006).

**Table 1:** Demographic and clinical profile of patients and controls.

	Patients	Controls
Gender (female/male)	19/22	19/22
Age (SD)	36,49 (10,87)	37,61 (10,79)
EDU (SD)	12,41 (3,32)	12,59 (2,65)
BDI (SD)	23,07 (11,89)	1,90 (3,07)
HAMD (SD)	11,54 (5,98)	–

*SD, standard deviation; EDU, years of education; BDI, Beck’s depression inventory score; HAMD, Hamilton depression scale score.*

**Table 2:** Patients’ demographic data and clinical profile.

Gender	Age	Diagnosis	Medication	Age of onset	Duration of illness
Female	57	F 32.1	Reboxetine, Citalopram	33	24
Female	54	F 33.1	Venlafaxine	50	5
Female	33	F 33.2	Venlafaxine	30	3
Female	34	F 32.1	Citalopram	24	10
Female	49	F 33.2	Venlafaxine, Quetiapine, Lithium carbonate	25	24
Female	33	F 33.3	Venlafaxine, Quetiapine	12	21
Female	33	F 32.1	Fluoxetine	33	1
Female	51	F 33.1	Agomelatine, Duloxetine	46	5
Female	27	F 32.2	Venlafaxine, Mirtazapine	21	6
Female	22	F 32.1	Venlafaxine	13	9
Female	26	F 32.1	Duloxetine	23	3
Female	31	F 33.1	Citalopram	11	20
Female	50	F 33.2	Duloxetine, Quetiapine	47	3
Female	38	F 33.1	Reboxetine	34	4
Female	30	F 32.1	“none”	30	1
Female	51	F 33.1	Escitalopram	31	20
Female	41	F 32.1	Venlafaxine	33	8
Female	29	F 32.2	Citalopram	29	1
Female	42	F 33.1	Mirtazapine	26	16
Male	45	F 32.1	Venlafaxine, Trimipramine	43	2
Male	55	F 33.2	Mirtazapine, Quetiapine, Duloxetine, Pipamperone	49	6
Male	37	F 33.1	Venlafaxine	35	2
Male	46	F 32.1	Venlafaxine, Opipramol	40	6
Male	52	F 32.1	Venlafaxine	44	8
Male	43	F 32.1	Mirtazapine	19	24
Male	27	F 32.1	Reboxetine	24	3
Male	25	F 32.1	Sertraline	21	4
Male	40	F 32.1	Venlafaxine	38	2
Male	30	F 33.1	Citalopram, Lithium	21	9
Male	38	F 32.1	Venlafaxine	35	3
Male	29	F 32.1	Lithium carbonate	24	5
Male	19	F 32.1	Citalopram	16	3
Male	28	F 33.2	Venlafaxine	27	1
Male	30	F 33.1	Opipramol, Sertraline, Mirtazapine	29	1
Male	53	F 33.2	Venlafaxine, Mirtazapine	44	9
Male	34	F 33.2	Venlafaxine, Quetiapine	25	9
Male	22	F 32.1	Venlafaxine	18	4
Male	41	F 33.1	Venlafaxine	36	5
Male	29	F 33.1	Bupropion, Quetiapine	20	9
Male	18	F 32.2	Remergil, Venlafaxine	17	1
Male	24	F 33.1	Citalopram	16	8

*Data for age of onset of depression and duration of illness is taken from self-reported information by the patients. Age, age of onset, and duration of illness are specified in years.*

### STIMULI

For a detailed description of stimulus material and procedure see Müller et al. (2011). In brief, the visual stimuli were color pictures obtained from the FEBA inventory (Gur et al., 2002) showing either a happy, neutral, or fearful facial expression. In total, 30 different faces were used, with five different female and five different male actors, each showing all three (happy/neutral/fearful) expressions. As pre-tests revealed that happy and fearful facial expressions were too clear in their emotionality to allow any contextual framing effects (Müller et al., 2011), they were made more ambiguous by merging them with the neutral mouths of the same actors. As auditory stimuli 10 laughs, 10 yawns, and 10 screams, each produced by five females and five males and lasting for 1500 ms, were used. Blurred versions of the neutral faces served as masks during the initial 1000 ms of sound presentation before the target faces were shown.

### AUDIOVISUAL PARADIGM

In total, 180 stimulus pairs, each consisting of a visual and an auditory stimulus, were used. Every face condition (happy/neutral/fearful) was paired with every sound condition (happy/neutral/fearful) resulting in a 3 × 3 design with nine different audiovisual conditions (fearful/scream, fearful/yawn, fearful/laugh, neutral/scream, neutral/yawn, neutral/laugh, happy/scream, happy/yawn, happy/laugh) and 20 individual audiovisual stimulus pairs per condition. The pairs were matched pseudo-randomly with regard to gender so that a female (male) face was always paired with a female (male) sound.

**FIGURE 1:**
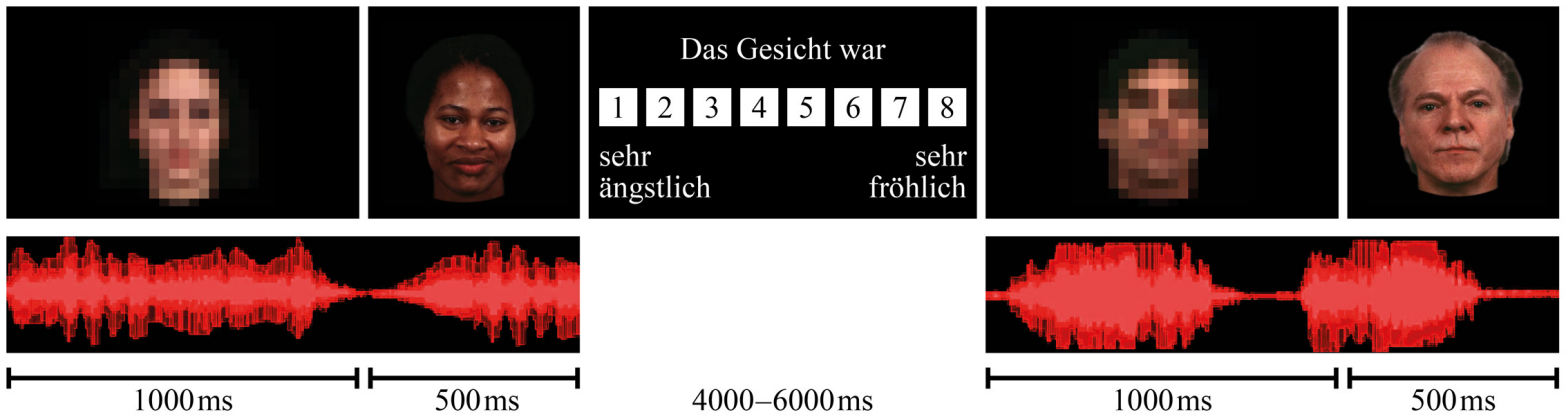
**Experimental procedure of the audiovisual paradigm.** Every trial started with a sound in combination with a blurred neutral face, after 1000 ms the screen switched to the target face, which was presented with the ongoing sound for another 500 ms.

**Figure 1** illustrates the experimental procedure. Every trial started with the presentation of a sound in combination with a blurred neutral face. After 1000 ms, the screen switched to a non-blurred picture of an emotional or neutral face (the target face), which was presented for another 500 ms with the ongoing sound. Participants had the task to ignore the sound and rate the facial expression on an eight-point rating scale (not including a neutral option and ranging from extremely fearful to extremely happy) as fast and as accurate as possible by pressing one of eight buttons on a response pad. To avoid expectation-led effects on the outcome of the experiment, the participants were told that the study focuses on attention processes. Stimuli were presented with the software Presentation 14.2 (http://www.neurobs.com/).

### UNIMODAL VALENCE AND AROUSAL RATINGS OF FACES AND SOUNDS

After the audiovisual paradigm, patients and controls rated emotional valence and arousal of all faces and sounds used in the audiovisual paradigm individually. For that, two separate runs were conducted, one for the unimodal facial expression rating and one for the unimodal sound rating. Both valence and arousal of the stimuli had to be rated on a 9-point rating scale, i.e., including a neutral option and ranging from very fearful to very happy/not at all arousing to very arousing.

### NEUROPSYCHOLOGICAL TESTING

To measure neurocognitive and psychomotor skills of patients and controls, diverse neurocognitive tests were conducted.

#### Visual attention/visuomotor speed

***Trail making tests (versions A and B)***. The two trail making tests [TMT-A and TMT-B; Army Individual Test Battery (AITB), 1944] were used to assess attention and visuomotor speed. Participants had the task to accurately connect as fast as possible (a) a consecutive sequence of numbers from 1 to 25 (TMT-A) and (b) a sequence of numbers from 1 to 13 mixed with the first 12 letters of the alphabet (TMT-B), respectively. The TMT-B, where numbers and letters had to be connected alternately, also assessed cognitive flexibility. Measurement for the test results was the time it took the participants to accomplish the task. Additionally we calculated difference scores between performance in TMT-A and TMT-B (TMT-Diff).

#### Motor speed/coordination

***Finger tapping test***. The finger tapping test was used to determine basic motor speed. Participants were asked to tap on the table as fast as possible for 10 s with their left or right index finger. This procedure was conducted three times for both (left and right) index fingers with short pauses in between to increase reliability but avoid muscular fatigue. For all six tapping runs, the number of taps was counted and the mean of all runs from both hands calculated (Halstead, 1947; Behrwind et al., 2011).

***Pointing movements.*** To assess basic motor coordination, the participants were asked to perform pointing movements with their left or right index finger (Defer et al., 1999). On the table in front of the subject, a 30 cm long horizontal line was marked and the task was to point on the two ends 10 times alternately as fast and as precise as possible. The time the participants needed to accomplish the task was measured. Again, this test was conducted three times for left and right index finger and the mean time of all six runs from both hands was calculated.

#### Crystalline intelligence

***Multiple choice vocabulary intelligence test (MWT).*** The multiple choice vocabulary intelligence test, version B (Lehrl, 1989), measured the participants’ crystalline intelligence. There were 37 rows of five words from which the participant had to choose the only actual word by ruling out four pseudo-words. The number of correctly detected words provided the test result.

#### Short-term and working memory

***Digit span subtest of the Wechsler Adult Intelligence Scale (Tewes, 1991).*** This test, in which verbally presented digit spans had to be repeated by the participant, consisted of two parts. In the first, used to measure short-term verbal memory and attention, the participant had to repeat the digit span in the same order as it was read to him (DS-F). For part two which assessed manipulation within working memory, the participant had to repeat the numbers backward (DS-B). For both test parts the number of correctly reproduced digit sequences was used as the test result.

Due to technical problems, the neuropsychological test results of one control subject could not be used and one patient did not take part in the TMT-A and TMT-B, while another one did not take part in the TAP10s and TAP10x30. The MWT was only conducted with native German speaking participants, and thus results from one of the patients and two control subjects are not available.

### STATISTICAL ANALYSIS

We analyzed our data using SPSS Statistics 21 (IBM). Most data except for those of the multimodal emotional processing task were not normally distributed and tests were individually chosen, adapted to the particular conditions. The threshold for significance was set at *p* < 0.05, Bonferroni-corrected for multiple comparisons if appropriate. Data from unimodal valence and arousal ratings of sounds and faces were analyzed with Mann–Whitney-*U* tests for group comparison and Wilcoxon-tests for comparisons between conditions (corrected for multiple comparisons). Data from the audiovisual paradigm were analyzed calculating a MANOVA (due to violation of sphericity) with the factors face, sound and group and the dependent variable emotional valence rating of faces. Significant main effects and interactions were furthermore analyzed with *post hoc t*-tests (corrected for multiple comparisons). To test for possible incongruence effects, two additional ANOVAS (for happy and fearful faces) with the factors congruence and group and the dependent variable emotional valence rating of faces were calculated. Significant findings were again further analyzed with *post hoc t*-tests (corrected for multiple comparisons). Furthermore, we calculated Spearman-rank-correlations between findings in emotional processing and Beck’s depression inventory score (BDI-scores) for the patient group.

Group differences in neurocognitive performance were tested with Mann–Whitney-*U* tests. Within the domains visual attention/visuomotor speed, motor speed/coordination and short-term/working memory, results were corrected for multiple comparisons (Bonferroni).

To investigate the relationship of emotional processing and neurocognitive functioning, we again calculated Spearman-rank-correlations.

## RESULTS

### UNIMODAL VALENCE AND AROUSAL RATING OF SOUNDS AND FACES

In total 12 Mann–Whitney-*U* tests were calculated (all results are Bonferroni-corrected for multiple comparisons). No significant group differences were found in the unimodal ratings of valence and arousal of faces and sounds (see **Table 3**).

**Table 3:** Group comparisons of unimodal valence and arousal ratings of sounds and faces – findings of Mann–Whitney-*U* tests.

		Arousal		Valence	
		*U*	*p*	*U*	*p*
Sounds	Positive	-2.018	0.044	-0.046	0.963
	Neutral	-0.191	0.848	-0.254	0.799
	Negative	-1.049	0.294	-0.599	0.549
Faces	Positive	-1.499	0.134	-0.803	0.422
	Neutral	-0.772	0.440	-1.545	0.122
	Negative	-0.603	0.546	-0.390	0.697

*No significant group differences were found (Bonferroni-corrected cut-off *p* < 0.017).*

Comparisons between conditions across groups demonstrated that happy and fearful faces were rated as more arousing than neutral ones and that all types of faces as well as all types of sounds differed from each other in their emotional valence rating (for mean values and SD see **Table 4**, all *p* < 0.017). In particular, fearful stimuli got the lowest ratings, followed by neutral faces and sounds, while the most positive ratings were given for happy stimuli.

**Table 4:** Mean values and SD for emotional valence and arousal ratings of faces and sounds.

	Happy (SD)	Neutral (SD)	Fearful (SD)
**Patients**
Emotional valence rating of faces	**7.049** (0.672)	**4.661** (0.536)	**3.437** (0.830)
Emotional valence rating of sounds	**7.742** (0.764)	**5.117** (0.483)	**1.649** (0.793)
Arousal rating of faces	**4.027** (1.521)	**3.698** (1.513)	**4.789** (1.635)
Arousal rating of sounds	**3.815** (1.640)	**3.129** (1.597)	**6.366** (2.095)
**Controls**
Emotional valence rating of faces	**6.917** (0.816)	**4.481** (0.583)	**3.527** (0.658)
Emotional valence rating of sounds	**7.707** (0.847)	**5.117** (0.785)	**1.576** (0.806)
Arousal rating of faces	**4.539** (1.865)	**3.963** (1.507)	**5.034** (1.417)
Arousal rating of sounds	**4.642** (2.022)	**3.061** (1.646)	**6.868** (1.837)

*There are no significant differences between patients and controls (Bonferroni-corrected cut-off *p* < 0.017).*

### AUDIOVISUAL PARADIGM

#### Valence ratings of faces in audiovisual setting

**FIGURE 2:**
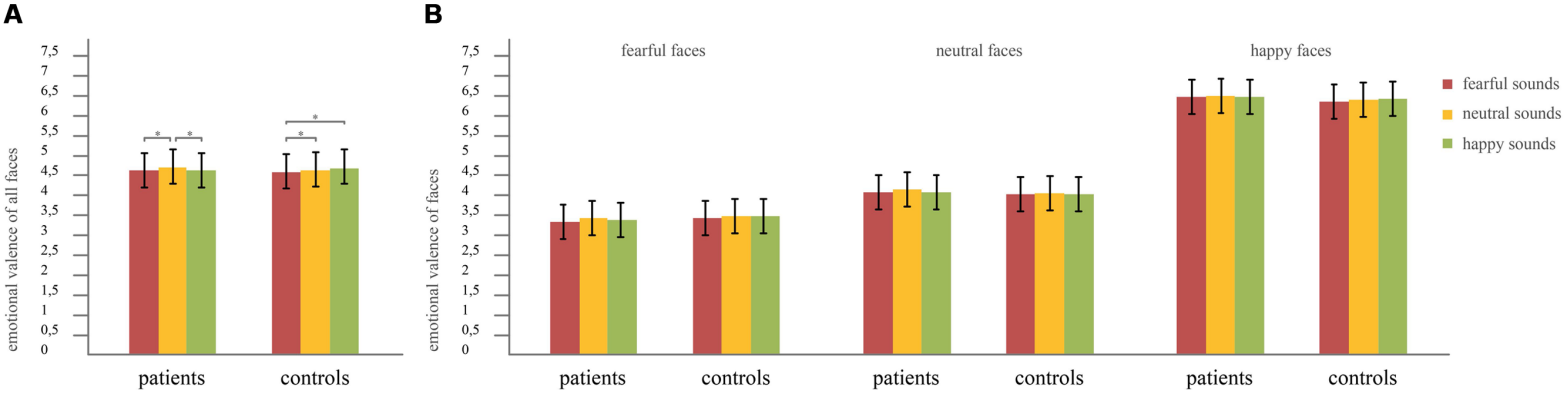
**Mean ratings of faces in the audiovisual setting.** Error bars indicate the SD of mean. Significant interaction group × sound. **(A)** Mean ratings for each sound condition across all face conditions. Asterisks indicate significant differences revealed by *post hoc* tests at *p* < 0.05, Bonferroni-corrected for multiple comparisons. Patients rated faces significantly more fearful when presented with fearful or happy sounds than when presented with neutral sounds, while controls rated faces significantly more fearful when presented with fearful sounds than when presented with neutral sounds, but ratings did not differ between the neutral and the happy sound conditions. **(B)** Mean values of the ratings of fearful, neutral, and happy faces separately. No interaction sound × face × group was found in the MANOVA, therefore no further *post hoc* tests were calculated.

A MANOVA with the factors face (happy/neutral/fearful), sound (happy/neutral/fearful) and group (controls/patients), and the dependent variable emotional valence rating was calculated. Multivariate testing was chosen due to violation of sphericity of sounds [χ^2^(2) = 6.267, *p* = 0.044] and faces [χ^2^(2) = 57.726, *p* < 0.001]. Assumptions of equality of error variances and equality of covariance matrices were met, indicated by non-significant Box’s *M* test and Levene’s tests. Results revealed significant main effects of sound (*F*_2,79_ = 12.89, *p* < 0.001, effect size: partial η^2^ = 0.25) and face (*F*_2,79_ = 401.81, *p* < 0.001, effect size: partial η^2^ = 0.91), but no main effect of group (*F*_1,80_ = 0.23, *p* = 0.63). Furthermore an interaction between sound × group could be found (*F*_2,79_ = 4.20, *p* = 0.018, effect size: partial η^2^ = 0.10), but no interactions of face × group (*F*_2,79_ = 1.91, *p* = 0.155), sound × face (*F*_4,77_ = 2.40, *p* = 0.058) or sound × face × group (*F*_4,77_ = 0.96, *p* = 0.437). To further analyze the significant interaction between sound × group *post hoc t*-tests were calculated (all results are Bonferroni-corrected for multiple comparisons). We found no significant global differences (across all types of concurrently presented faces) between groups, neither in the happy, nor neutral, nor fearful sound condition (faces paired with happy sounds: *T*_80_ = -0.065, *p* = 0.948; faces paired with neutral sounds: *T*_80_ = 0.861, *p* = 0.392; faces paired with fearful sounds: *T*_80_ = 0.602, *p* = 0.549). However, the *post hoc* tests demonstrated that while in healthy controls there was no difference in the rating of faces when concurrently hearing happy compared to neutral sounds (*T*_40_ = -0.08, *p* = 0.939), patients with depression rated faces as more fearful when presented with happy compared to neutral sounds (*T*_40_ = 4.61, *p* < 0.001). Furthermore, in controls the ratings of faces differed between the happy and fearful sound condition (*T*_40_ = -3.36, *p* = 0.002), whereas there was no difference between these two conditions in the patient group (*T*_40_ = -1.28, *p* = 0.209). Additionally, in both groups presentation of fearful compared to neutral sounds led to more fearful ratings of faces (controls: *T*_40_ = -3.09, *p* = 0.004; patients: *T*_40_ = -4.22, *p* < 0.001). These differences in the impact of sounds on the emotional valence ratings of faces between patients and controls are illustrated in **Figure 2**. For a more detailed analysis of the interaction, we further calculated difference scores in emotional valence ratings of faces between sound conditions, i.e., happy–neutral, happy–fearful, fearful–neutral. These difference scores were then compared between patients and controls using independent samples *t*-tests. The *t*-tests revealed a significant difference between patients and controls in the difference scores between the happy and neutral sound condition (*T*_80_ = -2.723, *p* = 0.008, effect size: Cohen’s *d* = 0.60). For patients, the mean value of the difference scores happy minus neutral was -0.068, indicating more fearful ratings of faces when presented with happy compared to neutral sounds. In contrast, the mean value of the respective difference scores in controls was 0.002, indicating only slightly happier ratings of faces when presented with happy sounds compared to neutral sounds. In contrast, neither the difference score of the happy minus fearful (mean values: patients 0.024; controls 0.077) nor the one of the fearful minus neutral sound condition (mean values: patients -0.092; controls -0.076) revealed any significant group differences (happy–fearful: *T*_80_ = -1.818, *p* = 0.073; fearful–neutral: *T*_80_ = -0.496, *p* = 0.621).

In summary, the main difference in face ratings between patients and controls was that patients rated faces in combination with happy sounds as more fearful than in combination with neutral sounds while controls did not.

Spearman-rank-correlations of difference scores between the happy and neutral sound conditions with BDI-scores were calculated for the patient group. Results did not reveal any significant associations (*r* = –0.087, *p* = 0.590).

#### Incongruence effect

**FIGURE 3:**
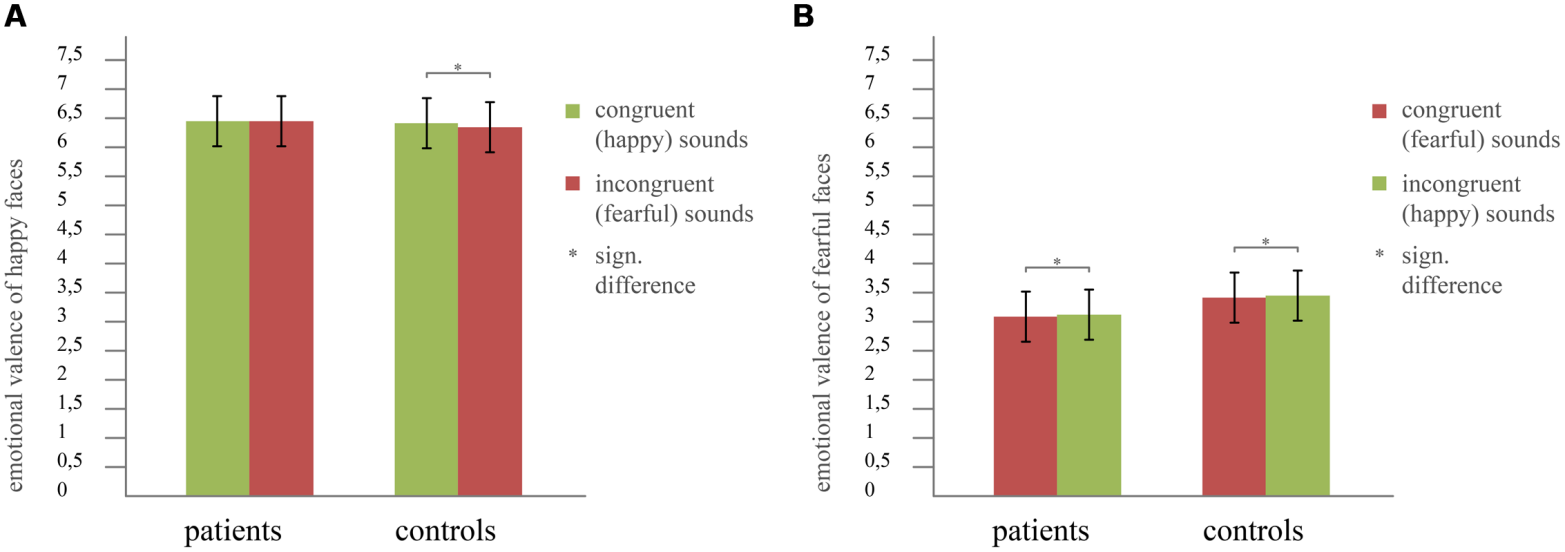
**Incongruence effect for **(A)** happy faces, revealing a significant interaction congruence × group and **(B)** fearful faces showing no group differences.** Error bars indicate the SD of mean. Asterisks indicate significant differences revealed by *post hoc* tests at *p* < 0.05, Bonferroni-corrected for multiple comparisons.

To investigate the interaction sound × group described above, we analyzed the audiovisual data more in detail. That is, we focused on the impact of emotional congruence/incongruence between concurrently presented sounds and faces on the valence ratings separately for happy and fearful faces. Therefore, we calculated two ANOVAs, one for happy faces and one for fearful faces. Both contained the factors congruence (congruent sound/incongruent sound) and group (controls/patients). In **Figure 3** the results are illustrated. For happy faces, we found a significant main effect of congruence (*F*_1,80_ = 5.40, *p* = 0.023, effect size: partial η^2^ = 0.06) and an interaction congruence × group (*F*_1,80_ = 6.15, *p* = 0.015, effect size: partial η^2^ = 0.07), but no main effect of group (*F*_1,80_ = 1.59, *p* = 0.211). In contrast, the ANOVA of fearful faces only revealed a significant main effect of congruence (*F*_1,80_ = 11.81, *p* = 0.001, effect size: partial η^2^ = 0.13) but neither a significant interaction congruence × group (*F*_1,80_ = 0.04, *p* = 0.834) nor a main effect of group (*F*_1,80_ = 1.23, *p* = 0.271). To further analyze the significant interaction between congruence × group for happy faces, we calculated *post hoc t*-tests (all results are Bonferroni-corrected for multiple comparisons). Those did not reveal any significant differences in emotional valence rating of faces between patients and controls, neither for the congruent nor for the incongruent condition (congruent: *T*_80_ = 0.76, *p* = 0.452; incongruent: *T*_80_ = 1.76, *p* = 0.083; see **Figure 3**). However, patients did not rate happy faces significantly different when paired with happy sounds (congruent condition) compared to fearful sounds (incongruent condition, *T*_40_ = -0.11, *p* = 0.910). In contrast, controls rated happy faces significantly happier when paired with happy sounds compared to fearful sounds (*T*_40_ = 3.29, *p* = 0.002; mean value happy sound: 6.35/mean value fearful sound: 6.24; see **Figure 3**).

### NEUROCOGNITIVE TESTS

#### Group comparisons

Mann–Whitney-*U* tests were calculated to compare the scores of the neurocognitive tests between the two groups (controls/patients). All results are Bonferroni-corrected for the number of tests within each test category. Significant differences between controls and patients were found for the TMT-A and TMT-B (TMT-A: *U* = -2.723, *p* = 0.006, effect size: *r* = 0.30; TMT-B: *U* = -2.492, *p* = 0.013, effect size: *r* = 0.29). In contrast, the other tests did not reveal any significant differences between groups (TMT-Diff: *U* = -0.385, *p* = 0.700; TAP10x30: *U* = -1.304, *p* = 0.192; TAP10s: *U* = -1.411, *p* = 0.158; DS-F:*U* = -1.215, *p* = 0.224; DS-B: *U* = -0.739, *p* = 0.460; MWT: *U* = -1.542, *p* = 0.123). **Table 5** shows the mean values and SD of all tests in patients and controls.

**Table 5:** Mean values and SD of the neuropsychological tests of all participants.

	*Patients:* mean value (SD)	*Controls:* mean value (SD)
*Visual attention/visuomotor speed*		
TMT-A** TMT-B** TMT-Diff	26.38 (15.61) 47.32 (19.94) 20.94 (14.47)	19.55 (5.68) 37.54 (11.48) 18.00 (9.43)
*Motor speed/coordination*		
TAP10s TAP10x30	101.66 (15.97) 16.76 (4.01)	107.01 (11.65) 15.63 (4.66)
*Short-term and working memory*		
DS-F DS-B	7.68 (1.93) 6.80 (1.79)	8.35 (1.98) 7.18 (1.82)
*Crystalline intelligence*		
MWT	27.68 (4.45)	29.50 (3.80)

**significant (*p* < 0.05, Bonferroni-corrected).

#### Correlations of findings in audiovisual emotional processing and neurocognitive functioning

To investigate the relationship between findings in audiovisual emotional processing and neurocognitive functioning in the patient group, Spearman-rank-correlations were calculated between neurocognitive test scores and the difference scores of the happy and neutral sound condition. All results are Bonferroni-corrected for the number of tests within each test category. No significant correlations between the difference scores and neurocognitive test scores were found (difference score between happy-neutral with: TMT-A: *r* = 0.326, *p* = 0.040; TMT-B: *r* = 0.235, *p* = 0.145; TMT-Diff: *r* = -0.096, *p* = 0.554; TAP10s: *r* = -0.30, *p* = 0.856; TAP10x30: *r* = -0.263, *p* = 0.101; DS-F: *r* = 0.076, *p* = 0.637; DS-B: *r* = -0.015, *p* = 0.928; MWT: *r* = 0.044, *p* = 0.787).

## DISCUSSION

The aim of the current study was to investigate emotional processing in a more naturalistic setting by adding an auditory context to visual stimuli, and furthermore the relationship between emotional and neurocognitive deficits in patients with depression. For that purpose, an audiovisual paradigm was conducted, where happy, fearful, and neutral faces had to be rated whilst ignoring concurrently presented emotional or neutral sounds. In addition, patients and controls completed diverse neurocognitive tests challenging attention, working memory, (visuo-)motor speed, coordination, and crystalline intelligence. Results in audiovisual emotional processing revealed an aberrant integration of happy sounds in major depression as patients rated faces significantly more fearful when combined with happy as compared to neutral sounds. Conversely, controls showed no significant differences between these two conditions. Findings in audiovisual emotional processing in patients did not correlate significantly with depressive symptom severity as indicated by BDI-scores. We only found significant group differences for two of the neurocognitive tests. Despite the fact that patients with depression were impaired both in audiovisual emotional processing and neurocognitive performance, we found no significant correlations between these two fields.

### UNIMODAL VALENCE AND AROUSAL RATINGS OF FACES AND SOUNDS

Several studies investigating unimodal emotional processing in patients with depression reported that patients showed a general bias toward more negative ratings of emotional and neutral stimuli such as faces, prosody, and music (Leppänen et al., 2004; Douglas and Porter, 2010; Naranjo et al., 2011) as well as an attentional bias toward negative emotional material (Leung et al., 2009; Milders et al., 2010). In the current study, however, we found no group differences with regard to valence and arousal ratings, neither for faces, nor for sounds. Apart from a general negative bias, these findings also contradict previous studies in depression, which reported respective group differences for unimodal stimuli (Csukly et al., 2009; Schlipf et al., 2013). They are rather in line with findings showing no group differences in unimodal emotional processing (Kan et al., 2004; Bourke et al., 2012; Müller et al., 2014). Müller et al. (2014) already suggested that such discrepancies might arise from differences in methodology like varying stimulus presentation times (Surguladze et al., 2004) or different emotions (fear vs. sadness) used as negative stimuli (Hu et al., 2012).

Thus, we would argue that the current findings suggest that, contradictory to our hypothesis, patients did neither show a general negative bias nor an emotional blunting with regard to unimodal ratings of emotional and neutral stimuli. This result, however, has to be taken with caution since differences in methodology (as mentioned above) clearly have an impact on the outcome of investigations regarding emotional processing.

### AUDIOVISUAL PROCESSING OF EMOTIONS IN PATIENTS WITH DEPRESSION

#### General group differences in multi-modal emotional processing

To date, only a few studies investigated audiovisual emotional processing in patients with depression. In our previous study (Müller et al., 2014), in which the same audiovisual task as in the current study was used, behavioral results revealed significant main effects of sounds and faces, but no main effect of group nor interactions. However, the further analysis in that paper indicated a deficit in patients when congruent emotional sound information had to be ignored. Since that previous sample was rather small and some of the reported behavioral findings only showed a trend toward significance, we here aimed to further investigate these effects within an extended sample. Overall, our results across groups are similar to that in the previous smaller sample, but now we found a significant sound × group interaction. This indicates that the impact of sounds on the perception (and therefore ratings) of faces was different in patients compared to controls. In particular, *post hoc* calculations in the current study showed that while controls rated faces quite similar in combination with neutral and happy sounds, patients rated faces significantly more fearful when combined with happy rather than neutral sounds. Further *t*-tests support this result by showing that the difference scores between the happy and neutral sound conditions differed significantly between patients and controls. These results thus highlight the aberrant integration and inhibition of irrelevant and especially positive auditory information in patients with depression. Correlational analyses, however, indicate that this effect is not related to symptom severity.

With regard to existing theories of emotional processing in depression, the current findings can be explained in different ways.

On the one hand, the sound × group interaction could be interpreted as a missing influence of concurrently presented positive auditory stimuli on the ratings of facial expressions in patients with depression. In line with this view, Surguladze et al. (2004) reported that patients exhibited a decreased tendency to interpret happy (but also neutral) faces as happy. Likewise Douglas and Porter (2010) described patients with depression as being less likely to interpret neutral faces as happy, while Loi et al. (2013) reported a reduced ability of patients to appraise positive stimuli of emotional body language. Importantly, however, our *post hoc* tests did not reveal group differences for any stimulus combination and rather indicated that patients differed in the ratings of faces paired with a positive sound compared to a neutral one while controls did not. Furthermore we only found this effect for the audiovisual processing task but not in the unimodal setting. Thus, the current results cannot support the view of an absent positive bias in patients with depression.

On the other hand, our results could also be explained in terms of a negative bias. In particular, for patients with depression, the current results show that any emotional sound (positive or negative) led to more negative ratings of concurrently seen emotional or neutral faces. This suggests that in depressed patients increased emotional input and higher arousal (cf. see Unimodal Valence and Arousal Rating of Sounds and Faces, emotional stimuli were rated as more arousing than neutral stimuli) received from two different channels generally leads to a more negative perception. Nevertheless, although fitting into the view of a more negative perception of emotions going along with depression (Gur et al., 1992; Bouhuys et al., 1999; Leppänen et al., 2004; Naranjo et al., 2011; Kaletsch et al., 2014), it has to be noted that this negative bias is limited to processing of emotions in an audiovisual setting (not for unimodal ratings of faces and sounds). Furthermore it only appears when facial stimuli are paired with happy sounds as healthy individuals are likewise negatively influenced by fearful sounds as patients are. Therefore the negative bias theory also does not explain the results sufficiently.

Yet our results can also be related to another more recent bias theory: Everaert et al. (2014) investigated the combined cognitive bias theory that has been reported for individuals with social phobia (Hirsch et al., 2006) in depression. They found that emotional biases in attention, interpretation, and memory in subclinical depression are strongly interrelated which potentially influences how daily life events are perceived. In particular, participants with higher depression scores paid more attention to negative emotional stimuli, made more negative interpretations and remembered negative material more frequently. When relating these findings to our results, it can be argued that patients paid special attention to fearful sounds and therefore held them in memory during the whole paradigm. Subsequently, their ratings of faces during concurrent presentation of laughter were then influenced by negatively biased memory of previous stimuli which led to a more fearful interpretation of faces. Although fitting with the impaired integration of happy sounds, this interpretation can, however, also not completely explain our findings. If the more fearful rating of faces during laughter had been due to maintenance of screams in memory, neutral sound presentation should have been influenced by this effect as well. However, our patients rated faces in combination with laughter significantly more fearful than in combination with yawning, and this was the only difference compared to controls.

In summary, none of the three presented bias theories can fully explain the current findings in patients with depression. Thus, our results suggest that in a multi-modal setting, impairments in emotional processing in depression cannot be reduced to a specific bias but are far more complex than previously thought.

#### Incongruence effects for happy and fearful faces

With reference to Müller et al. (2014) and in order to investigate the general effects found for audiovisual emotional processing in patients with depression more in detail, we specifically analyzed incongruence effects in emotional valence separately for happy and fearful faces. In particular, Müller et al. (2014) described a trend toward significance for the congruence × group interaction when analyzing the happy face condition, whereas they did not find any significant effect for the fearful face condition. Furthermore, their neuroimaging data revealed that for the congruent happy condition (happy face paired with happy sound), controls showed stronger deactivation in both left inferior parietal cortex (IPC) and left inferior frontal gyrus (IFG) compared to the patient group. Our current findings confirm the behavioral findings in a larger sample, thus providing increased power of results: patients with depression did not rate happy faces significantly different when paired with congruent (= happy) sounds in contrast to incongruent (= fearful) sounds, while controls rated happy faces significantly happier when paired with happy sounds than paired with fearful sounds. These behavioral findings fit well with the dysregulation of left IPC and IFG, which was found especially for the congruent happy condition (Müller et al., 2014). Thus, a failure to deactivate those two regions during processing of congruent positive audiovisual information in patients with depression might be associated with the missing behavioral incongruence effect found in the current study.

Our results regarding incongruence in emotional valence also shed further light on the effect found in the overall calculations described above (sound × group interaction) by showing that the effect of impaired integration of happy sounds is strongly connected to the happy face condition. Ratings of happy faces indeed became more negative in both patients and controls by incongruent (= fearful) sounds. However, when happy faces were paired with congruent (= happy) sounds, it seems that controls were able to inhibit or were positively influenced by these, while this was not the case in patients. Our findings hence suggest that patients are impaired in the inhibition of positive information in an additional sensory channel, especially when the target information is also positive. This additional presentation of positive information then has a negative impact on the ratings of the positive target emotion, what might reflect a tendency to perceive actually positive emotional input as threat.

Apart from the study by Müller et al. (2014), findings of multi-modal emotional processing in patients with depression are rare, but some studies exist, which investigate the impact of irrelevant (congruent/incongruent) emotional information on the perception and processing of emotional stimuli. Uekermann et al. (2008), for example, conducted several tests on identification and matching skills in depressed and healthy individuals, e.g., matching emotional/neutral prosody to semantics and faces. They reported deficits for all of these tests, except for conditions where information was congruent or in which sad stimuli were presented. These findings at first glance contradict the effect of impaired integration of congruent happy sounds in depression as found in the current study. However, this discrepancy may arise from the fact that the task in the current study was to rate the emotion of the faces on an intensity scale, while Uekermann et al. (2008) did not directly measure the influence of distracting information on perception. Rather they investigated differences in accuracy when matching/labeling concurrently presented information. Obviously patients are able to identify information from different channels as congruent, even when positive, but this does not necessarily mean that they perceive stimuli as equally positive as controls do.

Findings in the emotional Stroop task in depression shed further light on how irrelevant emotional information is processed by these patients. However, existing findings are quite inconsistent (Mogg and Bradley, 2005). A recent meta-analysis (Epp et al., 2012) thus quantified findings from behavioral studies investigating the (emotional) Stroop task and showed that depressed individuals exhibited a general attentional bias for emotional content, i.e., negative but importantly also positive words. With regard to positive stimuli, these findings are in line with the current results. The fact that we did not find an effect for the negative sound condition might again be due to the use of fearful rather than sad sounds as negative distractors (cf. see Unimodal Valence and Arousal Ratings of Faces and Sounds).

In summary, our results indicate that when confronted with audiovisual emotional information, patients with depression show in particular impairments when distracted by positive auditory information, especially when the visual information is also positive. This may – in line with studies showing that depression goes along with decreased responsiveness to reward (Henriques and Davidson, 2000) – suggest that depressed individuals are less likely to accept positive feedback as a kind of social reward from their environment, resulting in a more negative view on life and low mood.

### NEUROCOGNITIVE PERFORMANCE

Deficits in neurocognitive functioning in depression have been reported by numerous studies (e.g., Breslow et al., 1980; Fisher et al., 1986; Veiel, 1997; Zakzanis et al., 1998; Stordal et al., 2004). It is suggested that these deficits account for or at least substantially contribute to problems in everyday life like occupational functioning deficits, which are experienced by individuals with depression (Evans et al., 2013). However, findings on the pattern, extent and specificity of cognitive deficits in depression are quite heterogeneous (Ottowitz et al., 2002; Naismith et al., 2003; Marazziti et al., 2010; Lee et al., 2012; Quinn et al., 2012). For example, there is no agreement whether impairments are limited to executive functioning (Baune et al., 2012) or also relate to other domains like attention (Godard et al., 2012), psychomotor speed, visual learning/memory, and others (Lee et al., 2012). The current study revealed significant differences between patients and controls only in (both versions of) the trail making test. Conversely, there were no significant differences in any other neurocognitive test, contradicting the findings by Hoffstaedter et al. (2012) who conducted the same tests and reported group differences for most of them. Since motor speed was also assessed by TAP10s and Tap10x30, which both did not reveal group differences, our results indicate relatively specific deficits of visual attention and cognitive flexibility. Also, group differences in TMT-B in particular may point in the direction of a deficit in dealing with distracting information in patients with depression. These results highlight the importance of the specificity of assessment instruments for neurocognitive performance in depression (cf. Trivedi and Greer, 2014). Executive functioning, for example, may be operationalized and then measured by a large number of different tests and study designs. The ensuing results, however, would all be interpreted under the domain of executive functioning. This might explain heterogeneity in study findings. In addition, findings are also influenced by the patient sample investigated. The patient status (inpatient/outpatient) has, for example, an impact on the severity of impairments (Burt et al., 1995), possibly due to the fact that inpatients exhibit generally worse symptomatology (or rather, patients with worse symptoms are more likely to receive inpatient treatment). Furthermore, subtypes of depression also have to be considered when investigating neurocognitive performance (Naismith et al., 2003; Lee et al., 2012), as co-morbidities like anxiety disorder or bipolar disorder might have crucial impact on the outcome of neurocognitive test batteries. Thus, the fact that the patient group tested in the current study consisted exclusively of patients with unipolar depression who were free of co-morbidities may explain inconsistencies to some previous findings where patient groups were variably mixed. At last, antidepressant medication also plays a role, since medicated patients have been reported to perform better on neurocognitive tests than unmedicated ones (Gualtieri et al., 2006). Wagner et al. (2012) investigated changes in neurocognitive functioning during antidepressant treatment and found that performance of patients only improves in certain domains but not all. Especially test performance in TMT-B was excluded from improvement. This might provide an explanation for our results, as all but one patient of the current study were receiving antidepressant medication. However, subgroup analysis with regard to type of antidepressant medication was not possible due to the large variety in medication composition from patient to patient (see **Table 2**). Thus, future studies should deal with detailed analysis of the impact of certain antidepressant medication types on neurocognitive performance to be able to identify crucial factors on test results.

In summary, our findings show that when comparing patients with controls, differences are only found for attention and cognitive flexibility. This supports but also contradicts findings of other studies on neurocognitive functioning in depression and underlines the heterogeneity of depression.

### RELATIONSHIP BETWEEN AUDIOVISUAL EMOTIONAL PROCESSING AND NEUROCOGNITIVE FUNCTIONING

Emotional and neurocognitive aberrations have already been reported for depressed patients in numerous studies. However, only few have yet investigated how deficits in these two domains are related to each other. We thus correlated the effects found in the emotional processing task and the performance in the different neurocognitive tests and found that the effects revealed for audiovisual emotional processing, i.e., the difference of valence ratings of faces paired with happy compared to neutral sounds, were not related to performance in the neurocognitive tests. However, when not correcting for multiple comparisons, correlational analysis of the TMT-A test findings with impairments in emotional processing would reach significance. This is interesting, because the only two tests that revealed group differences were the two trail making tests. One could hence argue that impairments in the processing of interpersonal stimuli are possibly related to general deficits in visual attention. This would expand the findings of studies reporting selective visual attention for negative emotional material in depressed individuals (Eizenman et al., 2003; Kellough et al., 2008), indicating that attentional biases toward negative emotions alone cannot sufficiently explain impairments in emotional processing in depression. Rather, general attentional deficits might also lead to problems in concentrating on visual emotional material. This would fit with our finding that patients with depression are distracted more easily than healthy controls by additional irrelevant (especially positive) auditory stimuli. All in all, however, as this result did not survive correction for multiple comparisons, our findings nevertheless argue against a (direct) relationship between emotional processing and neurocognitive functioning. This contradicts findings of one previous study (Uekermann et al., 2008) reporting correlations between perception of affective prosody with inhibition abilities, set shifting, and working memory. On the other hand, another study (Langenecker et al., 2005) reported no correlations between impairments in emotional perception and neurocognitive performance and is hence well in line with our findings. Discussing their results, the authors mentioned that executive impairments are a feature of several different disorders, indicating that cognitive disturbances may not necessarily account for emotional impairments in depression. Likewise, Bourke et al. (2012) found that patients with depression perform significantly worse on verbal memory and spatial working memory tasks, but they did not find differences to healthy controls on unimodal emotional face recognition tasks (going in line with our findings in unimodal emotional processing). Even though they did not directly correlate emotional with cognitive performance, their results indicate that neurocognitive impairments are largely independent from emotional deficits.

Other studies (Everaert et al., 2014), however, reported associations of emotion perception and cognition in the sense of cognitive biases for negative emotions. In particular, they could show that emotionally biased cognitive processes like attention, interpretation and memory are highly interrelated with each other. Under this aspect, it has to be pointed out that it seems to make a huge difference if, on the one hand, general cognitive functions in absence of emotional material are examined or if, on the other hand, cognitive processing of emotional material is assessed. Thus, with regard to our findings, we can only infer that there is no relationship between general cognitive performance and impairments in emotional processing.

In summary, our results thus indicate that deficits in audiovisual emotional processing in depression seem to be widely independent from general neurocognitive functioning. Thus they do not support the assumption that deficits in emotional processing in patients with major depression are the results of impaired general attention or inhibitory functioning. Nevertheless we cannot completely rule out a bias component especially toward emotional material.

## SUMMARY

Our findings suggest that audiovisual integration of especially happy sounds is altered in patients with depression and that these alterations cannot be related directly to impairments in cognitive skills. Group differences in neurocognitive test performance were only revealed for measures of attention and cognitive flexibility. These results indicate that in real life, when emotions are processed in a multimodal fashion, deficits in depression cannot be reduced to an overall negative attitude toward emotional and neutral stimuli or a general absence of a positive bias. Rather, it is the influence of irrelevant positive stimuli, which plays a key role in emotion perception in depression. Though, impairments in audiovisual emotional processing do not change as a function of depressive symptom severity in patients. Furthermore there is no clear connection between emotional and neurocognitive impairments.

Although the current study did not directly investigate the role of attention in multisensory integration, our study adds further knowledge to this topic by investigating the relationship between both aspects in major depression and indicates that alterations in multi-modal emotional processing are not directly related to impaired attention.

## AUTHOR CONTRIBUTIONS

Veronika I. Müller and Simon B. Eickhoff designed the study, Veronika I. Müller acquired the data, Sophie Doose-Grünefeld and Veronika I. Müller analyzed the data, Sophie Doose-Grünefeld, Simon B. Eickhoff, and Veronika I. Müller wrote the paper.

## Conflict of Interest Statement

The authors declare that the research was conducted in the absence of any commercial or financial relationships that could be construed as a potential conflict of interest.
